# Imaging of viscoelastic soft matter with small indentation using higher eigenmodes in single-eigenmode amplitude-modulation atomic force microscopy

**DOI:** 10.3762/bjnano.9.103

**Published:** 2018-04-06

**Authors:** Miead Nikfarjam, Enrique A López-Guerra, Santiago D Solares, Babak Eslami

**Affiliations:** 1 Department of Mechanical Engineering, University of Maryland, College Park, MD 20740, USA; 2 Department of Mechanical and Aerospace Engineering, The George Washington University, Washington, DC 20052, USA

**Keywords:** higher eigenmodes, multifrequency AFM, soft matter, viscoelasticity

## Abstract

In this short paper we explore the use of higher eigenmodes in single-eigenmode amplitude-modulation atomic force microscopy (AFM) for the small-indentation imaging of soft viscoelastic materials. In viscoelastic materials, whose response depends on the deformation rate, the tip–sample forces generated as a result of sample deformation increase as the tip velocity increases. Since the eigenfrequencies in a cantilever increase with eigenmode order, and since higher oscillation frequencies lead to higher tip velocities for a given amplitude (in viscoelastic materials), the sample indentation can in some cases be reduced by using higher eigenmodes of the cantilever. This effect competes with the lower sensitivity of higher eigenmodes, due to their larger force constant, which for elastic materials leads to greater indentation for similar amplitudes, compared with lower eigenmodes. We offer a short theoretical discussion of the key underlying concepts, along with numerical simulations and experiments to illustrate a simple recipe for imaging soft viscoelastic matter with reduced indentation.

## Introduction

Since the invention of atomic force microscopy (AFM), researchers have sought to increase the number of observables that are recorded during a single-pass measurement, as well as improve the sensitivity with which those observables are recorded [1–5]. In an effort to control the sensitivity and versatility of the instrument, it has been proposed to use higher cantilever eigenmodes, either by themselves in single-eigenmode imaging [6–9] or within multifrequency techniques [10]. For example, in the original multifrequency AFM method, introduced by Garcia and coworkers and known as bimodal AFM [4], the first eigenmode of the cantilever is excited using the AM-AFM method and used for measuring topography, while a higher eigenmode (generally the second eigenmode) is simultaneously excited in “open loop” (with constant drive amplitude and frequency) to map the surface properties of the material via the phase channel of the eigenmode. An extension of this method, known as trimodal AFM, adds a third eigenmode to modulate tip indentation, thus enabling characterization of the subsurface for certain types of soft samples [5]. As we discussed in that introductory work, in general, the use of higher eigenmodes can be helpful for the purpose of increasing tip–sample indentation since the sensitivity of the eigenmode to the tip–sample forces decreases as the product of the force constant of the eigenmode times its amplitude (*k_i_A_i_*) increases [5]. In that work we also offered the general statement that, for a fixed oscillation amplitude, higher eigenmodes are expected to generate greater indentation into the sample due to their higher force constant (e.g., *k*_2_ ≈ 39*k*_1_). However, this is only true when no rate-dependent effects (i.e., viscous effects) are present. When such effects are present, the outcome is not always obvious to predict a priori. This is because higher eigenmodes also have higher frequencies (e.g., *f*_2_ ≈ 6.27*f*_1_), which lead to higher tip velocities for a given value of the oscillation amplitude. This, in turn, results in a faster sample deformation, which in a viscoelastic material causes larger reaction forces that oppose the downward motion of the tip into the sample. These larger forces cause greater perturbation of the cantilever oscillation, reducing its ability to indent the sample [11].

This paper explores the above competing effects for single-eigenmode imaging with higher eigenmodes. After briefly discussing the key theoretical concepts, we present numerical and experimental results involving the use of AM-AFM with the first eigenmode, AM-AFM with the second eigenmode, and bimodal AFM using the first two eigenmodes, and offer a simple guideline for the characterization of soft viscoelastic matter with small indentation. The paper is written in a very brief manner, focusing only on the above competing effects, in order to single out this useful concept for the small-indentation imaging of viscoelastic materials.

## Theoretical considerations regarding sample indentation

### Effect of the product *kA* on sample indentation

As has been described in previous studies [5,12–13], the equation of motion for a given eigenmode can be written in a dimensionless fashion as,

[1]
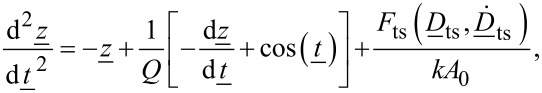


where *A*_0_ is the free oscillation amplitude, *z*(*t*) = *z*(*t*)/*A*_0_ is the dimensionless tip position with respect to the cantilever base position, *D*_ts_(*t*) = *D*_ts_(*t*)/*A*_0_ is the dimensionless tip–sample distance, 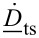 is the dimensionless tip–sample velocity, and *t* = ω_0_*t* is the dimensionless time, and the approximation *A* ≈ *A*_0_ = *F*_0_*Q*/*k* has been used, where *A* is the free oscillation amplitude of the cantilever and *F*_0_ is the amplitude of the oscillatory excitation force. This equation indicates that the relevance of the tip–sample forces to the eigenmode dynamics can be diminished or magnified by adjusting the product *kA*_0_. Thus, if the forces are not dependent on the tip velocity, higher eigenmodes will lead to greater sample indentation for a fixed value of the oscillation amplitude *A*_0_, due to their higher force constant. An implicit qualitative conclusion is that similar sensitivity should be observed during single-eigenmode AM-AFM for different eigenmodes, *i* and *j*, when *k_i_A_i_* = *k_j_A_j_*. Again, it is important to stress that this qualitative trend is only expected to hold if the tip–sample force is independent of velocity.

### Effect of tip velocity on sample indentation

**Figure 1:**
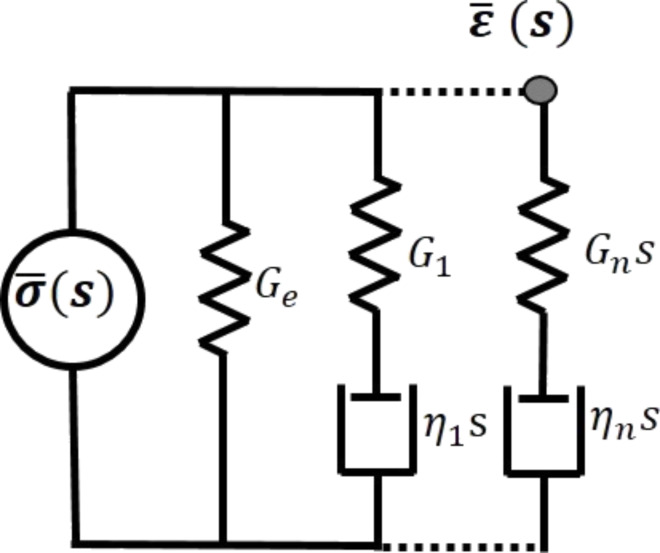
Generalized Maxwell or Wiechert mechanical model diagram representing the relationship between stress and strain in the complex plane for a linear viscoelastic material with multiple characteristic times. This model describes arrheodictic (there is no steady-state flow) behavior. *G_n_* refers to the modulus of the *n*-th spring. η*_n_* refers to the viscosity of the *n*-th dashpot. *G_e_* refers to the rubbery modulus. The Laplace transformed stress 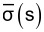 is regarded as the excitation and the transformed strain 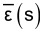
as the response.

Viscoelastic materials generally exhibit two extremal behaviors depending on whether they are probed at very high loading rates (high tip velocities) or at very low loading rates (low tip velocities). Consider a material described by the generalized Maxwell mechanical model for viscoelastic materials, shown in Figure 1, where the material response is modeled using a combination of elastic springs and viscous dashpots [13–14]. When the material is probed at infinitely low loading rates, the springs in the Maxwell arms do not experience any deformation at all because the dashpots yield (recall that the force exerted by the dashpots is proportional to the deformation velocity), and the only element ruling the mechanical behavior is the rubbery modulus (*G_e_* spring). At this extreme, no energy dissipation takes place and the material behaves in a soft-elastic manner [11,15–17]. On the other hand, when the material is probed at extremely high loading rates, the dashpots do not deform and the behavior of the mechanical model is ruled by the summation of all the individual springs in parallel. In this case, the material behaves in a stiff-elastic manner, without any energy being dissipated, and the response is ruled by the glassy modulus of the material (*G_g_*):

[2]
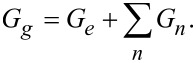


In general, the response of the material to the oscillation of the tip tapping on its surface falls in between these two extremes, and approaches one or the other behavior according to how relatively fast or slow the material is deformed. For the specific case of an AFM probe tapping on a viscoelastic surface, one may expect that when it is probed with the second eigenmode instead of the fundamental eigenmode, where the former has a natural frequency that is approximately 6.27 times the fundamental eigenfrequency, the material will behave in a regime closer to the stiff-elastic behavior. That is, the material will exert larger opposing forces when it is impacted by the tip, which makes it more capable of perturbing the eigenmode oscillation for a given product *kA*_0_, as the rightmost term in Equation 1 will become more prominent due to a larger numerator. Generally speaking, a stiffer material has the potential to cause greater reductions in the eigenmode oscillation amplitude with smaller indentations.

## Results and Discussion

### Numerical results

A numerical study was performed simulating a parabolic tip penetrating a polyisobutylene half-space. The dynamics of the cantilever tip are assumed to be mainly contained in the lower modes and therefore we included only the contribution of the first three flexural eigenmodes, using an individual equation of motion for each of them, all coupled through the tip–sample forces:

[3]
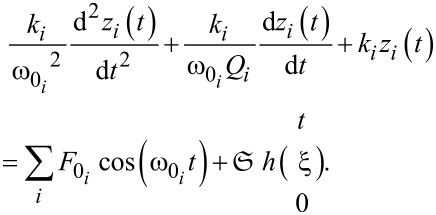


Here *z_i_*, *k_i_*, *Q_i_* and 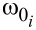 refer to the *i*-th (with *i* = 1, 2, 3) eigenmode displacement, cantilever stiffness, cantilever quality factor, and resonance frequency, respectively. The summation term on the right-hand side refers to the excitation force applied, where *F*_0_ is the amplitude of the *i*-th term in the oscillatory excitation force. The notation employed to represent the tip–sample force term in Equation 3,


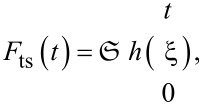


emphasizes the nature of the viscoelastic material modeled. According to it, the tip–sample force is a functional of the sample deformation *h*, i.e., the force at the current time *t*, *F*_ts_(*t*), depends on the history of the surface deformation at all previous times ξ, from ξ = 0 to ξ = *t*. This definition of tip–sample force emphasizes the history-dependent behavior of the material, therefore the tip–sample force not only depends on tip position but also on tip velocity and higher displacement derivatives, in addition to force derivatives [11,16].

This contact-mechanics problem for viscoelastic half spaces has been formulated by independent studies [18–21], which agree that during the loading portion (monotonically increasing tip–sample contact radius) the relationship between force and displacement is given by:

[4]
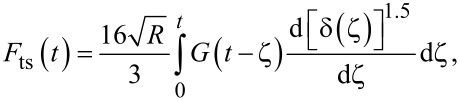


where ζ is a dummy variable used to perform the convolution integral, *F*_ts_ is the tip–sample contact force, *R* is the radius of curvature of the tip apex, δ is the tip indentation and *G*(*t*) is the shear relaxation modulus, which in our case is described by the Generalized Maxwell (also called Wiechert) model (see Figure 1):

[5]
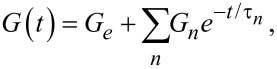


where τ*_n_* = η*_n_*/*G_n_* is the ratio between viscosity (η*_n_*) and modulus (*G_n_*) in the *n*-th arm in the model in Figure 1. The values for *G_n_* and τ*_n_* used in the simulations were digitalized from the data provided by Brinson and Brinson [17], who obtained the values by fitting the experimental data of Catsiff and Tobolsky [22]. The digitalized values are summarized in Table 1. The plot for these values is also provided in Supporting File 1 (Figure S1).

**Table 1:** Generalized Maxwell parameters for poly-isobutylene given by Brinson and Brinson [17].

element number	relaxation time 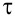 (s)	modulus (Pa)

1	1.166 × 10^−9^	4.132 × 10^8^
2	4.852 × 10^−9^	8.227 × 10^8^
3	2.250 × 10^−8^	6.315 × 10^8^
4	9.652 × 10^−8^	3.607 × 10^8^
5	3.832 × 10^−7^	1.533 × 10^8^
6	1.671 × 10^−6^	4.522 × 10^7^
7	7.196 × 10^−6^	2.230 × 10^7^
8	2.888 × 10^−5^	6.101 × 10^6^
9	1.479 × 10^−4^	2.606 × 10^6^
10	5.871 × 10^−4^	1.108 × 10^6^
11	2.361 × 10^−3^	2.816 × 10^5^
12	9.355 × 10^−3^	1.288 × 10^5^
13	4.028 × 10^−2^	6.354 × 10^4^
14	1.798 × 10^−1^	7.212 × 10^3^
15	8.160 × 10^−1^	1.336 × 10^4^
16	3.293	9.276 × 10^4^
17	1.303 × 10^1^	4.567 × 10^4^
18	5.847 × 10^1^	1.315 × 10^5^
19	2.967 × 10^2^	8.110 × 10^4^
20	1.046 × 10^3^	1.390 × 10^5^
21	5.278 × 10^3^	1.068 × 10^5^
22	2.635 × 10^4^	1.276 × 10^5^
23	8.797 × 10^4^	6.263 × 10^4^
24	4.124 × 10^5^	3.094 × 10^4^
25	1.831 × 10^6^	1.384 × 10^−1^
26	7.757 × 10^6^	1.322 × 10^−1^

The contact mechanics described by Equation 4 are strictly only valid for the approach portion of the indenter trajectory. A generalized approach has been derived by Ting, which is applicable for any arbitrary (a priori) known loading history [21]. In our simulations, where a priori knowledge of the loading history is not available, we use an alternative approach based on the method of dimensionality reduction (MDR) in which a three-dimensional continuum is replaced by a uniquely defined one-dimensional linear viscoelastic foundation [23]. This simple method has proven to generate exact solutions for the general viscoelastic problem [24–25], and we therefore employ it in our simulations. For details about the simulations refer to code provided in [26].

**Figure 2:**
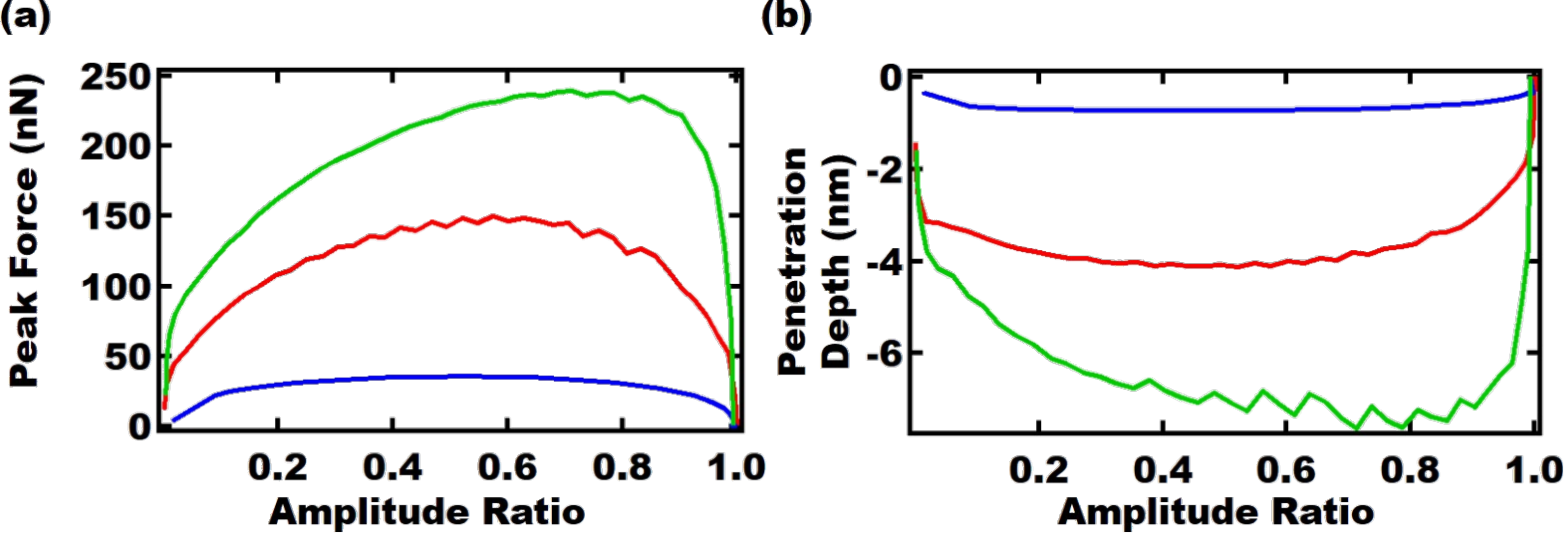
Numerical simulations corresponding to a parabolic AFM tip tapping on a polyisobutylene surface, described as a viscoelastic material containing multiple characteristic times using the generalized Maxwell model with the parameters of Table 1. The results show: (a) the peak tip–sample interaction force, and (b) the maximum indentation depth, with respect to amplitude setpoint ratio for AM-AFM using the first eigenmode (red line), AM-AFM using the second eigenmode (blue line), and bimodal AFM using the first two eigenmodes (green line). *f*_1_ ≈ 45 kHz, *f*_2_ ≈ 280 kHz, *k*_1_ ≈ 5.80 N/m, *k*_2_ ≈ 210 N/m, *A*_1_ ≈ 350 nm, *A*_2_ ≈ 11 nm. The parameters are selected based on the experimental values found by tuning the cantilever as discussed in the next section. The amplitudes provided are the free oscillation amplitudes.

**Figure 3:**
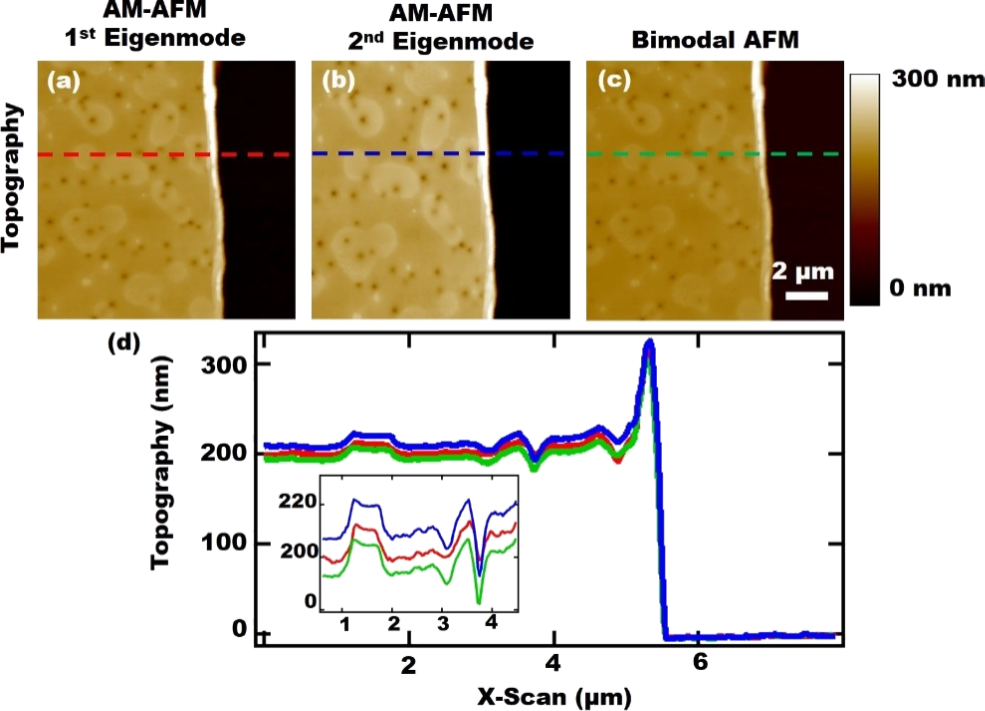
Polystyrene thin-film topography images for AM-AFM using the fundamental eigenmode (a), AM-AFM using the second eigenmode (b), and bimodal AFM using the first two eigenmodes (c); (d) shows the scan line profiles, whereby the color code on the graph is based on the dashed lines in the images. The inset graph is the enlarged graph of a portion of the topography in order to show the differences in topography values among the three different experiments. *f*_1_ ≈ 45 kHz, *f*_2_ ≈ 280 kHz, *k*_1_ ≈ 5.80 N/m, *k*_2_ ≈ 210 N/m, *A*_1_ ≈ 350 nm, *A*_2_ ≈ 11 nm, amplitude setpoint (on the controlled amplitude) = 50%. For all the experiments the oscillation amplitudes were selected such that *k*_1_*A*_1_ ≈ *k*_2_*A*_2_. The setpoint was selected such that the experiments remained in the repulsive regime.

Three different AFM schemes where used in the simulations, namely AM-AFM with the fundamental eigenmode, AM-AFM with the second eigenmode, and bimodal AFM using the first two eigenmodes. In all cases, the product(s) *k_i_A_i_* of the active eigenmode(s) was/were kept constant. Figure 2a presents the peak force observed during the cantilever trajectory as a function of the setpoint ratio of the modulated amplitude. Figure 2b presents the indentation depth as a function of the setpoint ratio of the modulated amplitude. As the results show, AM-AFM using the second eigenmode has the smallest penetration depth. On the other hand, bimodal AFM leads to the greatest tip penetration, since there are “*k_i_A_i_*” contributions from two eigenmodes. The kinks in these non-smooth curves may be ascribed to energy transfer occuring between eigenmodes [27], especially for the case of second-eigenmode AM-AFM operation using large setpoints. This led us to use lower setpoints in the experimental results (see below in Figure 3) in order to minimize this phenomenon. To model the dynamics of the cantilever, a system of three ordinary differential equations was used, in which each equation corresponds to one eigenmode of the cantilever (assuming the dynamics are mainly contained in the first three eigenmodes) [28]. The equations are solved numerically as described in previous studies [29] and details can be found in the computational code provided in [26].

### Experimental results

Polystyrene thin film height measurements were performed using the same imaging modes as in the numerical simulations. All three measurements were performed with a single cantilever having *f*_1_ ≈ 45 kHz and *k*_1_ ≈ 5.80 N/m. Polystyrene with 33 kDa molecular weight diluted to 2.5 wt % in THF was used, which was spin-coated onto a silicon wafer at 1400 rpm for 60 s. We have selected a polystyrene of low molecular weight to prepare a sample that displays time-dependent behavior within the deformational timescale in our studies (previous studies have quantified the dependence of characteristic times on the molecular weight for polystyrene [30]). In order to ensure a homogenous surface, the relative humidity was controlled to be approximately 30% during the spin-coating process. A portion of the polymer film was scratched off from the substrate to provide a reference for the thickness measurements. All of the measurements were performed on the same location on the polymer sample and the results are provided in Figure 3. As in the numerical simulations, AM-AFM with the second eigenmode led to the smallest indentation (largest measured film thickness) compared to the other two experiments, and the relative indentations for AM-AFM using the fundamental eigenmode and bimodal AFM follow the trend observed in the numerical results for polyisobutylene. The discrepancy between the magnitude of the indentation in the simulations (Figure 2) and the experiments (Figure 3), i.e., the larger indentation in the experiments, may be explained partly by viscoelastic steady-state flow induced by the tip during the experiments, through which the surface may not fully recover after the tip taps on it, differences in tip geometry, and differences in surface material properties.

## Conclusion

As stated in the Introduction, we have focused on the simple concept of the key competing effects governing tip–sample indentation in the characterization of soft viscoelastic materials, providing qualitative mathematics that can help the experimentalist select imaging conditions that place the various eigenmodes in roughly equal footing (i.e., via comparisons of their *k_i_A_i_* product). Clearly, the conclusions and guidelines presented here are only general and can vary in applicability from sample to sample for a number of reasons. For example, (i) the arguments made based on Equation 1 rely on the order of magnitude of the various terms, and the quality of the approximation *A* ≈ *A*_0_ = *F*_0_*Q*/*k* decreases as the amplitude setpoint is decreased. Additionally, (ii) not all soft materials are equally viscoelastic. Some samples may be more or less viscous or more or less elastic than others, so the balance of the competing effects governing indentation may shift in one or the other direction. Furthermore, (iii) the environment in which the sample is imaged plays a key role. In particular, many additional effects occur in liquid environments, which we have not considered in this study, but where many soft samples are imaged. Some of these effects include mass loading of the cantilever, excitation of higher cantilever eigenmodes, and the inability to accurately track the tip motion for piezoelectrically excited cantilevers [31–33]. Nevertheless, as our results show, it may in many instances be possible to reduce tip–sample indentation of soft materials by using higher eigenmodes during their AFM characterization, keeping in mind the amplitude adjustments based on matching the product *k_i_A_i_* of different eigenmodes and taking advantage of the deformation-rate-dependence of viscoelastic materials. We encourage further research in this area, especially in liquids, where the softest (biological) samples find their native environment.

## Supporting Information

File 1 Prony coefficients for polyisobutylene.
