# Supplementary material for: Imaging of viscoelastic soft matter with small indentation using higher eigenmodes in single-eigenmode amplitude-modulation atomic force microscopy
Source: Beilstein J Nanotechnol. 2018 Apr 6;9:1116–22. doi: 10.3762/bjnano.9.103 (PMC5905250; doi:10.3762/bjnano.9.103)
Supplement: File 1 — Prony coefficients for polyisobutylene. [file Beilstein_J_Nanotechnol-09-1116-s001.pdf]

Supporting Information

for

Imaging of viscoelastic soft matter with small indentation using higher eigenmodes in single-eigenmode amplitude-modulation atomic force microscopy

Miead Nikfarjam[1], Enrique A. López-Guerra[2], Santiago D. Solares[2] and Babak Eslami*[1]

Address: [1]Department of Mechanical Engineering, University of Maryland, College Park, MD 20740, USA and [2]Department of Mechanical and Aerospace Engineering, The George Washington University, Washington, DC 20052, USA

Email: Babak Eslami* - beslami@umd.edu

* Corresponding author

Prony coefficients for polyisobutylene

Viscoelastic properties of polyisobutylene

The following plot describes the modulus (Pa) of Polyisobutylene versus different relaxation times (in seconds). This figure is a graph based on the Prony coefficients provided in Table 1, highlighting the region of interest for this study.

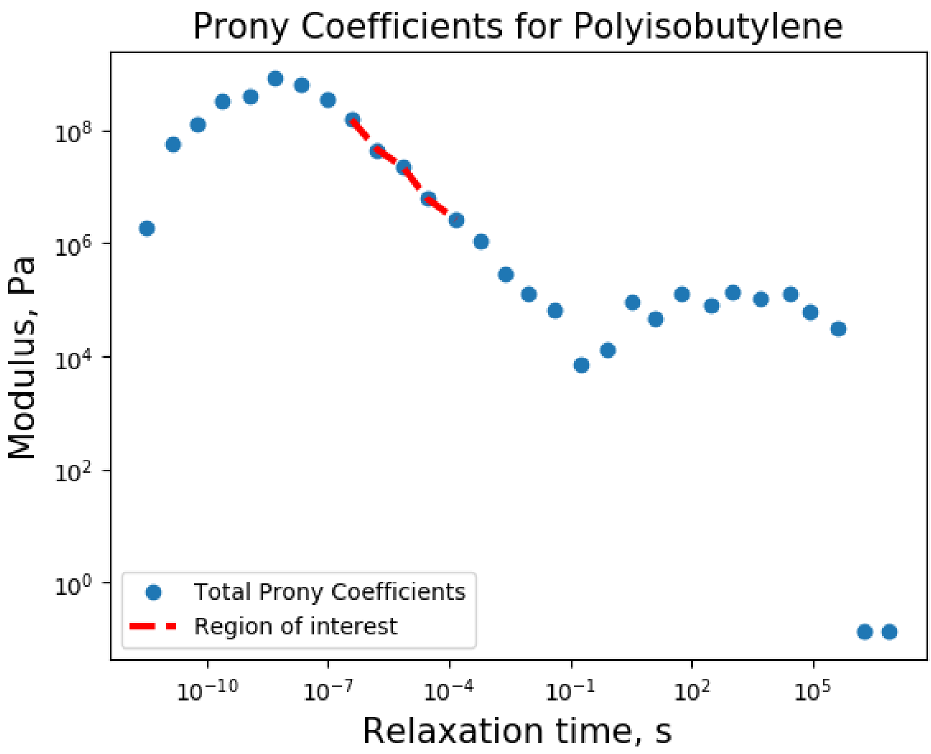

Figure S1: Prony Coefficients for polyisobutylene: modulus (Pa) as a function of relaxation time (s).
